# Therapeutic Potential of Exploiting Autophagy Cascade Against Coronavirus Infection

**DOI:** 10.3389/fmicb.2021.675419

**Published:** 2021-05-14

**Authors:** Subhajit Maity, Abhik Saha

**Affiliations:** School of Biotechnology, Presidency University, Kolkata, India

**Keywords:** coronaviruses (CoVs), SARS-CoV-2, COVID-19, autophagy, virophagy

## Abstract

Since its emergence in December 2019 in Wuhan, China, severe acute respiratory syndrome coronavirus 2 (SARS-CoV-2) created a worldwide pandemic of coronavirus disease (COVID-19) with nearly 136 million cases and approximately 3 million deaths. Recent studies indicate that like other coronaviruses, SARS-CoV-2 also hijacks or usurps various host cell machineries including autophagy for its replication and disease pathogenesis. Double membrane vesicles generated during initiation of autophagy cascade act as a scaffold for the assembly of viral replication complexes and facilitate RNA synthesis. The use of autophagy inhibitors - chloroquine and hydroxychloroquine initially appeared to be as a potential treatment strategy of COVID-19 patients but later remained at the center of debate due to high cytotoxic effects. In the absence of a specific drug or vaccine, there is an urgent need for a safe, potent as well as affordable drug to control the disease spread. Given the intricate connection between autophagy machinery and viral pathogenesis, the question arises whether targeting autophagy pathway might show a path to fight against SARS-CoV-2 infection. In this review we will discuss about our current knowledge linking autophagy to coronaviruses and how that is being utilized to repurpose autophagy modulators as potential COVID-19 treatment.

## Introduction

Coronaviruses (CoVs) belong to the subfamily *Orthocoronavirinae* in the family *Coronaviridae*, are a large group of enveloped viruses. There are four genera within the subfamily *Orthocoronavirinae* that includes Alphacoronavirus (αCoV), Betacoronavirus (βCoV), Gammacoronavirus (γCoV) and Deltacoronavirus (δCoV) (Fehr and Perlman, 2015). The viral genome is single-stranded positive-sense RNA, ranges from 26-32 kb and is the largest among all known RNA viruses (Fehr and Perlman, 2015; Su et al., 2016). While αCoV and βCoV infect mammals and have been shown to be responsible for diseases in humans, γCoV and δCoV infect only birds (Chan et al., 2013). Previously, two viral epidemics caused by βCoVs have been reported. In 2002, an outbreak of severe acute respiratory syndrome (SARS) was first detected in the Guangdong province of southern China (Zhong et al., 2003; Zhao, 2007) and according to WHO it subsequently affected 26 countries resulted in approximately 8000 cases with a mortality rate of 11%. In recent past in 2012, Middle East respiratory syndrome (MERS) was first reported in Saudi Arabia (Zaki et al., 2012) and later spread to 27 different countries, with a mortality rate of roughly 35% (WHO –update). In both of these outbreaks, the origins of the βCoVs are not fully understood however, according to the analysis of different viral genomes, it has been suggested that these viruses may have originated in bats and subsequently were transmitted to other intermediate animal hosts sometime in the distant past before infecting humans. For example, the Himalayan palm civet (*Paguma larvata*) harbors SARS-CoV and the dromedary camel (*Camelus dromedarius*) harbors MERS-CoV (Cui et al., 2019; Chan et al., 2020).

The ongoing outbreak of coronavirus disease 2019 (COVID-19) caused by the severe acute respiratory syndrome coronavirus 2 (SARS-CoV-2) creates a serious threat to public health and economy worldwide (Lu et al., 2020; Zhu et al., 2020). The first case of COVID-19 was reported in December 2019 in Wuhan, China and in spite of tremendous efforts to contain the disease, the infection quickly spread across the world (Zhu et al., 2020). Accordingly, in January 30, 2020 the World Health Organization (WHO) declared it a ‘Public Health Emergency of International Concern (PHEIC) and eventually on March 11, 2020 a ‘global pandemic’. As of now, almost 136 million cases have been registered including around 3 million deaths in more than 200 countries and territories^1^. COVID-19 disease includes a range of clinical symptoms from mild fever with dry cough, sore throat and breathing discomfort after an average incubation period of 1 week to SARS leading to pneumonia, pulmonary edema, lung damage, and failure of several vital organs such as liver, kidney, and heart. Importantly, a significant number of the infected population remains asymptomatic, making the spread of the disease more uncontrollable (Huang et al., 2020; Jin et al., 2020; Zhu et al., 2020). Patients suffering from severe SARS-CoV-2 infection require mechanical ventilator support system in intensive care units (Jin et al., 2020).

Owing to unavailability of a proper vaccine or specific anti-viral drugs, there is an urgent and desperate need to find drugs that blocks initial infection and restricts viral spread to control the COVID-19 pandemic situation. Given the overwhelming scenario of the disease, a number of potential therapeutic approaches against SARS-CoV-2 infection have been envisaged and are currently being investigated. Recent studies have implicated autophagy machinery in SARS-CoV-2 replication and pathogenesis (Cortegiani et al., 2020a,b; Gao et al., 2020; Wang et al., 2020). In this review, we will discuss the existing knowledge regarding the interplay between SARS-CoV-2 together with other CoVs and autophagy pathway, with an aim of finding potential targets for therapeutic interventions in order to restrain CoV infections.

## Autophagy – a Catabolic Cytoprotective Cell Mechanism

Autophagy is cellular catabolic mechanism that arbitrates the degradation of undesirable protein aggregates and damaged organelles and recycles the degraded components. First elucidated in yeasts, autophagy is evolutionarily conserved in higher forms of life, which helps in maintaining the cell homeostasis and clearance of invading intracellular pathogens as well (Dikic and Elazar, 2018). There are three types of autophagy - microautophagy, macroautophagy and chaperone-mediated autophagy (CMA) (Mizushima and Komatsu, 2011). In response to growth factor deprivation, macroautophagy is induced resulting in the initiation and formation of double membrane vesicles (DMVs), also known as autophagosomes, where the cytoplasmic cargos are enclosed and subsequently delivered to the lysosomes for degradation. Lysosomal degradation leads to generation of basic building blocks such as free fatty acids and amino acids, which thereafter are released into the cytoplasm for recycling (Xie and Klionsky, 2007). In contrast, microautophagy is morphologically different than macroautophagy as it does not involve formation of distinct autophagy related structures - autophagosomes. Little is known about its functional details and its possible role in human health and disease. In this process, cytoplasmic cargos are directly delivered to the lysosome and subsequently degraded (Mijaljica et al., 2011). CMA has currently only been observed in mammalian cells and is highly specific process, which selectively utilizes Heat Shock Protein Family A (Hsp70) Member 8 (HSPA8) and KFERQ motif to bind substrate-protein and deliver it into the lysosome for degradation (Kaushik and Cuervo, 2012). Among the three types of autophagy, macroautophagy has been studied in detail and hereafter referred to as autophagy.

**FIGURE 1:**
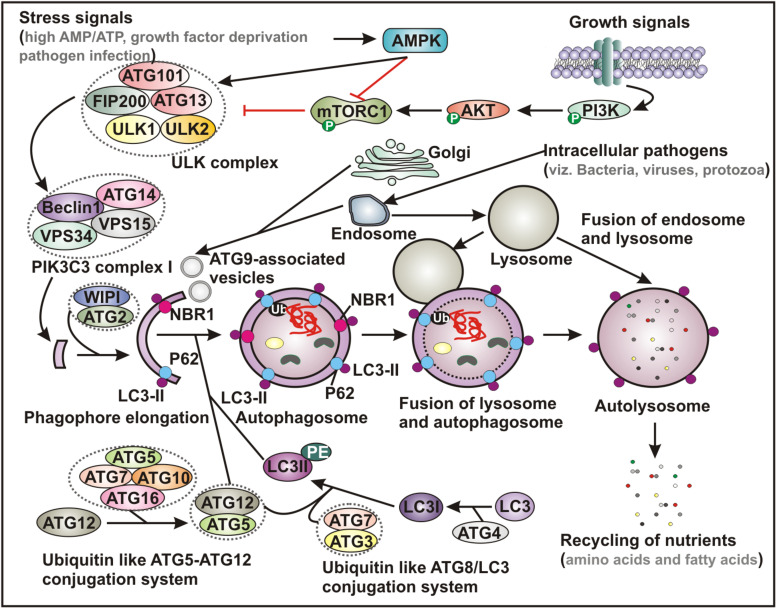
Schematic representation of basic autophagy pathway. Upon deprivation of growth factors, increase in AMP level and pathogen infection lead to AMPK activation and subsequent inhibition of mTORC1 function. In contrast, in the presence of growth signals PI3K-AKT signaling pathway activates mTOR. Inhibition of mTORC1 results in activation of ULK complex, which phosphorylates Beclin-1, leading to VPS34 activation and initiation of phagophore formation. ULK functions in a complex with ULK1, ULK2, FIP200, ATG13 and ATG101, while VPS34 function within the PIK3C3 complex containing its regulatory subunit, VPS15, ATG14 and Beclin-1, which further recruits to WIPI and ATG2 for phagophore elongation. Several ATG proteins engage two evolutionarily conserved ubiquitin-like conjugation systems - ATG12-ATG5 and phosphatidylethanolamine (PE)-conjugated LC3 (LC3-II) targeted to the pre-autophagosomal membrane. In the ATG12-ATG5 conjugation system, the complex further interacts with ATG16, where ATG7 functions as an E1-like enzyme and ATG10 factions as an E2-like enzyme. In the other system, LC3 is first cleaved by a cysteine protease ATG4 to generate LC3-I, which is further conjugated with PE to form membrane bound LC3-II facilitated by ATG7 and ATG3. The cytoplasmic damaged cargo is then ubiquitinated, captured by adaptor molecules - p62 or NBR1 and subsequently delivered to the phagophore membrane. Matured autophagosome then fuses with endolysosomal vesicles forming an autolysosome, where the cargo is degraded and provide nutrients. AMPK, AMP activated protein kinase; mTORC1, mammalian target of rapamycin complex 1; PI3K, phosphatidylinositol 3-kinase; PIK3C3, Phosphatidylinositol 3-Kinase Catalytic Subunit Type 3; LC3, microtubule-associated protein 1 light chain 3; Ub, ubiquitin; NBR1, neighbor of BRCA1 gene 1.

Many molecular mechanisms have been explored to reveal the basic processes underlying autophagy. Overall, autophagy process is initiated by a dedicated cohort of proteins encoded by autophagy-related-genes (ATG*s*), which are activated and recruited to the isolated membranes (Figure 1). Functionally, mammalian ATG proteins can be sub-categorized into six clusters, involved in different steps during autophagosome biogenesis - vesicle nucleation, membrane elongation and completion. Six categories of ATG proteins include (i) the ULK complex, a serine-threonine kinase complex containing ULK1, ULK2, ATG13, FIP200 and ATG101; (ii) the phosphatidylinositol 3-phosphate class III lipid kinase complex I containing PIK3C3/VPS34, PIK3R4/VPS15, Beclin-1 and ATG14; (iii) the phosphatidylinositol 3-phosphate PtdIns3P-binding WIPI/ATG18–ATG2 complex; (iv) the multi-spanning transmembrane protein ATG9A; along with two ubiquitin-like conjugation complexes - (v) the ubiquitin-like ATG5-ATG12 system, which conjugates ATG12 to ATG5 in association with ATG16L1, ATG7 and ATG10 and (vi) the ubiquitin-like ATG8/LC3 conjugation system, which ensures the lipidation of the Atg8 family members in association with ATG7 and ATG3 (Figure 1). There are six members in mammalian Atg8 family, which includes MAP1LC3A, MAP1LC3B, MAP1LC3C, GABARAP, GABARAPL1, and GABARAPL2 (Glick et al., 2010; Dikic and Elazar, 2018).

The autophagy process can be either non-specific where it engulfs the cytoplasmic cargo in bulk, or it can also selectively target distinct cellular targets through utilizing specific adapter-proteins that interact with both the substrates and the autophagosomal membranes (Farre and Subramani, 2016; Zaffagnini and Martens, 2016). Recognition of a given intracellular component or damaged organelle by these adapter-proteins - p62/sequestosome-1 or neighbor of breast cancer 1 (NBR1) is triggered by the ubiquitin tags. While the ubiquitin-binding domain (UBD) of a specific adapter protein identifies the ubiquitin moiety conjugated with the cytoplamsic cargo, the LC3-interacting region (LIR) directs binding of LC3 proteins attached to the autophagosomes (Figure 1; Farre and Subramani, 2016; Zaffagnini and Martens, 2016). Accordingly, the expanding list of specific clearance of various endogenous substrates by this pathway includes removal of damaged mitochondria (mitophagy) (Youle and Narendra, 2011), peroxisomes (pexophagy) (Germain and Kim, 2020), chloroplast turnover in plants (chlorophagy) (Izumi et al., 2019), endoplasmic reticulum (reticulophagy or ER-phagy) (Wilkinson, 2019), and ribosomes (ribophagy) (An and Harper, 2018), degradation of protein aggregates (aggrephagy) (Yamamoto and Simonsen, 2011) and lipid droplets (lipophagy) (Kounakis et al., 2019) as well as intracellular pathogens including fungi, bacteria and viruses (xenophagy) (Sharma et al., 2018).

Although a number of physiological roles have been implicated through clearance of cellular wastes, upsetting the natural balance of autophagy mechanism can result in pathological outcome (Mizushima and Komatsu, 2011; Dikic and Elazar, 2018; Sharma et al., 2018). Upon nutrient starvation, endoplasmic reticulum (ER)-stress, hypoxia, impaired intracellular cholesterol trafficking, rapid declines in trophic factors or hormones as well as infectious pathogens, autophagy-induction renders a cytoprotective effect. Moreover, accumulating evidence indicates the critical role of autophagy in many human diseases, such as cardiovascular diseases, cancers, neurodegenerative diseases and numerous metabolic disorders (Yamamoto and Simonsen, 2011; Ahmad et al., 2018; Dikic and Elazar, 2018). Among these, the implication of autophagy in viral infection (virophagy) has been extensively investigated and highly appreciated (Ahmad et al., 2018; Choi et al., 2018; Sharma et al., 2018). Figure 1 describes the general autophagy pathway involving various cell molecules that assist in initiation and biogenesis of autophagosome, convergence with endocytic pathway and degradation of and recycling of the captured intracellular cargo.

## Autophagy and Viral Infections

Studies investigating the critical interplay between autophagy and viruses demonstrated that many viruses have evolved strategies to escape autophagy-mediated degradation and occasionally even utilize autophagy mechanism to facilitate their own replication. Consequently, several autophagy-modulating agents have shown anti-viral potentials and are currently being pursued for therapeutic interventions (Tovilovic et al., 2014; Galluzzi et al., 2017; Bowman et al., 2018; Gao et al., 2020; Wang et al., 2020).

## Impact of Autophagy on Coronaviruses

**FIGURE 2:**
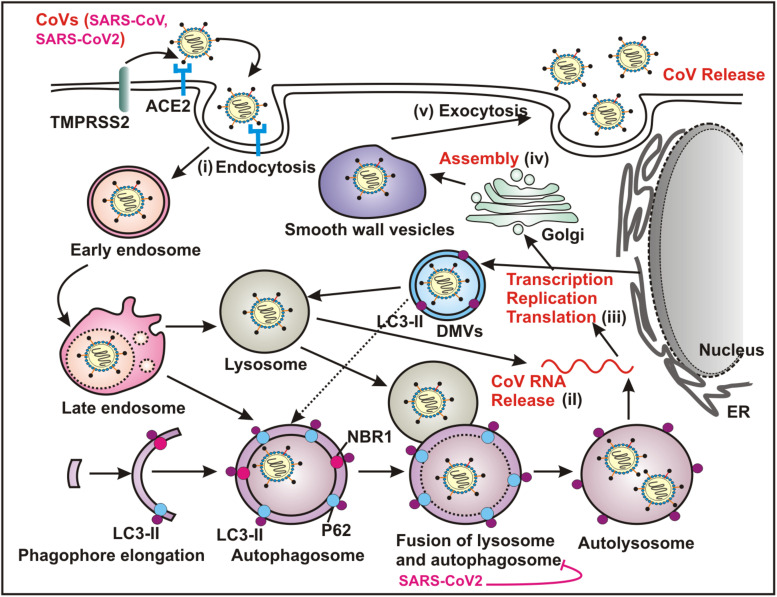
Coronaviruses (CoVs) utilize autophagy pathway for replication. Both SARS-CoV and SARS-COV-2 recognize ACE2 as the cellular surface receptor that mediates the viral entry into the host via endocytosis. Several CoVs utilize ATG5/ATG7 independent autophagy that establishes an endocytic pathway-Golgi route potentially interacting with the multiple stages of virus replication cycle - (i) entry mechanism by endocytosis and fusion of viral and host membranes; (ii) uncoating and releasing the viral genomic RNA; (iii) transcription, replication and translation; (iv) trafficking and assembly and (v) egress by exocytosis. The replication transcription centers are intimately associated with DMVs, which are generated from the ER. DMVs mimic autophagosomes and subsequently these structures fuse with the late endosome, and the lysosome, which results in degradation of the sequestered cytoplasmic cargo. A number of CoVs including SARS-CoV-2 specifically block the fusion between autophagosome and lysosome. ACE2, angiotensin converting enzyme 2; ER, endoplasmic reticulum; DMVs, double membrane vesicles.

Although, the precise link between autophagy and CoV infection remains largely unclear, current knowledge indicates that CoVs interact with the multiple components of autophagy machinery in order to facilitate viral replication (Figure 2). The most obvious clue signifying the connection between autophagy and CoV pathogenesis is the induction of characteristic DMVs in both cases (Blanchard and Roingeard, 2015; Choi et al., 2018; Du Toit, 2020). The formation of RNA-replication complexes of CoVs occur at DMV and apparently generation of infectious viral-particles of a number of CoVs rely on these ER-derived membranes (Du Toit, 2020).

The first report supporting the participation of autophagy in CoV replication was based on mouse hepatitis virus (MHV) (Prentice et al., 2004a; Jackson et al., 2005). The authors evidently demonstrated that upon infection, MHV induces formation of DMVs, resembling autophgosomal structures, which in turn assist viral replication by providing a comfortable niche (Ulasli et al., 2010). The connotation of DMVs and so as autophagy in MHV replication was concluded by the observation of co-localization of the viral replication complexes at DMVs and several important the autophagy proteins including LC3 and ATG12 as well as impaired viral replication in ATG5 knockout cells (Prentice et al., 2004a). Likewise, the same group also demonstrated co-localization of several pivotal replication-proteins of SARS-CoV with LC3, further validating the notion of autophagy involvement in CoV replication (Prentice et al., 2004b). In contrast, further studies demonstrated that replication of both MHV and SARS-CoV is not utterly reliant on autophagy, as deletion of key regulatory genes required for autophagososme biogenesis, such as ATG5 and ATG7 failed to impair virus replication (Zhao et al., 2007; Reggiori et al., 2010; Schneider et al., 2012). Moreover, autophagy induction by growth factor deprivation also did not appreciably affect MHV replication (Schneider et al., 2012). Taken together, these results indicate that induction of DMVs upon MHV infection might not directly engage autophagy pathway, conversely based on the evidence of co-localization of specific viral replication proteins like NSP2 and NSP3 with LC3 (Reggiori et al., 2010); it is also difficult to conclusively rule out the likely involvement of autophagy in MHV replication. The induction of DMV and MHV replication are perhaps two distinct processes except they both utilize LC3 molecule.

Pharmacological inhibition or genetic ablation of autophagy machinery demonstrated that replication of another CoV, the transmissible gastroenteritis virus (TGEV), is inhibited by autophagy (Guo et al., 2016), while on the contrary, another study reported mitophagy plays a proviral mechanism in TGEV infection by counteracting oxidative stress and apoptosis (Zhu et al., 2016). In addition, a different group showed that a key viral replication transmembrane protein NSP6 encoded by another CoV infectious bronchitis virus (IBV) can promote autophagosome formation, while by disrupting autophagy flux it significantly diminishes the delivery of viral particles in the lysosomal compartments for degradation (Cottam et al., 2011). Intriguingly, this property is also shared by NSP6 encoded by mammalian CoVs including MHV and SARS-CoV (Cottam et al., 2011). It has been suggested that CoV NSP6 proteins promote omegasome and autophagosome formation independently of starvation, without direct involvement of mTOR inhibition and induction of ER stress (Cottam et al., 2011). However, recently NSP6 of another CoV porcine epidemic diarrhea virus (PEDV) was shown to activate autophagy via downmodulating PI3K/AKT/mTOR axis (Lin et al., 2020).

Previously, it has been shown that NSP3 proteins encoded by a number of CoVs including SARS-CoV, HCoV-NL63 and PEDV contain papain-like protease (PLP) domains exhibiting immunomodulatory roles (Devaraj et al., 2007; Clementz et al., 2010; Xing et al., 2013). Later, ectopic expression of membrane bound PLP2 encoded by HCoV-NL63 and PEDV and PLpro-TM encoded by SARS-CoV and MERS-CoV were shown to obstruct autophagy flux by blocking the fusion between autophgaosomes and lysosomes (Chen et al., 2014). PLP2 was found to interact with LC3 and Beclin-1, and Beclin-1 depletion partially reversed the inhibition of PLP2-mediated innate immune signaling cascade, representing a potential therapeutic approach to control CoV infection (Chen et al., 2014). In agreement to these, MERS-CoV infected hepatocytes also demonstrated inhibitory effects on autophagy by selective activation of the ERK/MAPK and PI3K/AKT/mTOR pathway and upon pharmacological inhibition, MERS infection is inhibited (Kindrachuk et al., 2015). In addition, another study suggested that MERS-CoV infection results in Beclin-1 degradation by recruiting Skp2 E3 ligase activity and hinders the fusion between autophgaosomes and lysosomes (Gassen et al., 2019). Induction of autophagy by genetic or pharmacological ablation of Skp2 activity can drastically diminish MERS-CoV replication, providing a potential drug target for CoV infection through modulating autophagy mechanism (Gassen et al., 2019). In this article, the authors evidently demonstrated that overexpression of three viral proteins including NSP6, p4b and p5 resulted in autophagy inhibition (Gassen et al., 2019). As discussed above, NSP6 negatively regulates autophagy flux (Cottam et al., 2011), p4b has phosphodiesterase function and by inhibiting RNAse L activation it activates autophagy (Gusho et al., 2016), while p5 is located in the ER-Golgi intermediate compartment and was previously shown to inhibit IFN-β induction (Yang et al., 2013), which may represent another link to autophagy. However, the functional relevance of both p4b and p5 linking to autophagy warrants further investigation.

Overall, accumulating evidence suggests potential inhibitory regulation by multiple CoVs on autophagy mechanism (Table 1). For example, as similar to IBV and MERS-CoV, recently a preprint article demonstrated that the novel SARS-CoV-2 infection in bronchial epithelial cells (NCI-H1299) and monkey kidney cells (VeroFM) inhibits autophagy flux by limiting the activation of AMP-protein activated kinase (AMPK) and mammalian target of rapamycin complex 1 (mTORC1), and thereby protecting viral particles from subsequent lysosomal degradation and assisting in replication process (Gassen et al., 2020). Moreover, SARS-CoV-2 infection leads to downregulation of autophagy-inducing spermidine, and AKT1/SKP2-mediated proteasomal degradation of Beclin-1 (Gassen et al., 2019). Therefore, by specifically targeting these pathways using aliphatic polyamine spermidine as dietary supplement and/or combination of AKT inhibitor and Beclin-1 stabilizing agent could result promising outcome in COVID-19 patients. Likewise, another preprint article also demonstrated that SARS-CoV-2 infection in lung carcinoma cell lines (A549 and Calu3) blocks autophagy flux and thereby contributing to enhanced viral replication (Singh et al., 2020). Through profiling host global gene transcriptional changes using both cell lines as well as clinical samples, Singh et al. demonstrated that inflammatory response, mitochondrial fission, and autophagy cascade were specifically perturbed in SARS-CoV-2 infected cells as compared to MERS-CoV and Influenza A virus (Singh et al., 2020). Specifically, SARS-CoV-2 infection was shown to block autophagic flux by elevating GSK3β expression in lung cancer lines, or by depleting autophagy genes, such as SNAP29 (Synaptosomal-Associated Protein 29) along with lysosomal acidification genes in COVID-19 lung biopsy samples (Singh et al., 2020). SNAP29 is involved in mediating membrane fusion between autophagosomes and lysosomes (Bernard and Klionsky, 2015; Diao et al., 2015). Thus, conceivably drugs specifically inducing autophagic flux might exhibit potent anti-viral activities in COVID-19 patients.

**TABLE 1:** Interplay between autophagy and coronaviruses (CoVs).

**CoVs**	**Interaction with autophagy machinery**	**Consequences in viral pathogenesis**	**References**
MHV	NSP2 and NSP3 colocalize with endogenous LC3; MHV infection promotes non-canonical autophagy by inducing ER-derived DMVs	DMVs and autophagosomal membranes serve as sites for viral replication; ATG5 and LC3 knockdown decrease MHV replication	Prentice et al., 2004a; Reggiori et al., 2010
SARS-CoV	Viral infection as well as co-expressions of NSP3, NSP4 and NSP6 induce DMV formation; NSP6 colocalizes with LC3 and generates ER-mediated autophagosomes via an omegasome intermediate	Autophagy increases viral replication	Prentice et al., 2004b
MERS-CoV	Viral infection as well as co-expressions of NSP3 and NSP4 induce DMV formation; activates ERK/MAPK and PI3K/AKT/mTOR signaling networks; viral infection induces Skp2 E3 ligase mediated Beclin-1 degradation; blocks autophagy flux	Autophagosome formation helps in viral replication and by blocking autophagy flux it bypasses autophagy-mediated degradation and subsequent antigen presentation	Kindrachuk et al., 2015
IBV	Although IBV does not induce autophagy pathway, NSP6 colocalizes with LC3 and constricts its puncta pattern and thus limiting the size of autophagosomes; NSP6 induces ER-derived autophagosome formation through an omegasome intermediate	Induction of autophagosome formation enhances viral replication	Cottam et al., 2011
HCoV-NL63	NSP3 induces autophagosome formation, but blocks autophagy flux	Autophagy increases viral replication	Chen et al., 2014
PEDV	Viral infection enhances autophagosome formation and; NSP6 activates autophagy via PI3K/AKT/mTOR axis	Autophagy increases viral replication; ATG5 and Beclin-1 knockdown decrease PEDV replication	Lin et al., 2020
TGEV	Viral infection blocks autophagy	ATG5, ATG7 and LC3 knockdown increase TGEV replication	Guo et al., 2016
SARS-CoV-2	Viral infection inhibits autophagy flux by downmodulating AMPK/mTORC1 activation; hampers autophagy flux by upregulating GSK3β, or by downregulating p62 and SNAP29 genes	Reduces autophagosome lysosome fusion efficiency and thereby activates viral propagation	Gassen et al., 2020; Singh et al., 2020

*DMV, double membrane vesicle; EAV, equine arteritis virus.*

Collectively, thus far, a universal role of autophagy in CoV replication could not be established. As described in Table 1, the incongruities of results by multiple groups arose possibly due to utilization of different CoVs tested in different cells along with employment of different techniques to study autophagy. Nevertheless, further in depth investigation is warranted to precisely elucidate the role of autophagy in CoV replication and thereby allowing development of novel therapeutic strategies modulating autophagy cascade.

## Autophagic Modulators as Potential Treatment of CoV Infection

Due to the incredible variability of viral proteins, researchers greatly paid attention into host cell-encoded pathways for therapeutic intervention against viral infection. A growing body of evidence suggests autophagy as a pivotal component of virus-host interaction and represents as impending antiviral target (Bowman et al., 2018; Choi et al., 2018). One of the key determining factors in viral infection is the entry of the virus into the host cells. Based on the current knowledge, it is generally believed that most of the CoVs including MHV, SARS-CoV and MERS-CoV engage the endocytic pathway as one of the key entry mechanisms into various host cell types (Yang and Shen, 2020). Although, at present, there is no direct evidence demonstrating the new SARS-CoV-2 also exploits the endocytic pathway as entry mechanism, it utilizes angiotensin converting enzyme II (ACE2) receptor as similar to SARS-CoV and moreover is susceptible to the inhibitory effect of chloroquine (CQ), an anti-malarial drug that specifically increases lysosomal acidic pH and thereby blocking autophagy flux and endosomal function (Kuba et al., 2005; Wang et al., 2020; Zhou et al., 2020a). Therefore, a number of therapeutic approaches focusing on modulating autophagy-endocytic pathway have been investigated and are currently under clinical trials to control the ongoing devastating pandemic situation of COVID-19 worldwide (Bonam et al., 2020; Gao et al., 2020; Van Dorn, 2020). In the later section, we will discuss about such studies and potential therapeutic interventions of multiple autophagy modulators against COVID-19 disease (Table 2).

**TABLE 2:** Inhibitory effects of autophagy modulators on coronaviruses.

**Drugs**	**Mechanism of actions linking autophagy**	**Impact on Coronavirus**	**References**
Chloroquine* and Hydroxychloroquine*	Impede with autophagy function by escalating the endosomal/lysosomal pH	Block endocytosis mediated entry mechanisms, uncoating and exit (exocytosis) of PEDV, SARS-CoV, MERS-CoV, HCoV-OC43 and SARS-CoV-2; does not inhibit SARS-CoV-2 infection specifically in lung cells expressing low CTSL expression	Keyaerts et al., 2004, 2009; Mauthe et al., 2018; Hoffmann et al., 2020b; Liu et al., 2020; Schrezenmeier and Dorner, 2020; Wang et al., 2020
Rapamycin/Sirolimus*	Activates autophagy by inhibiting mTORC1	Reduces infection of MERS-CoV, PEDV and SARS-CoV-2	Dowling et al., 2010; Kindrachuk et al., 2015; Gassen et al., 2020; Lin et al., 2020
GW5074/Sorafenib*	Blocks autophagy by inhibiting c-Raf	Inhibits MERS-CoV infection	Kindrachuk et al., 2015; Prieto-Dominguez et al., 2016
Reserpine*	Inhibits autophagy flux by blocking autolysosome formation	Inhibits SARS-CoV replication	Wu et al., 2004; Lee et al., 2015
Nitazoxanide*	Stimulates autophagy by blocking mTORC1	Inhibits replication of MERS-CoV and SARS-CoV-2	Wang et al., 2018, 2020
Niclosamide*	Promotes autophagy by blocking mTORC1	Inhibits viral antigen synthesis of SARS-CoV and MERS-CoV	Gassen et al., 2019; Wang et al., 2019
Everolimus*	Induces autophagy by blocking mTORC1	Inhibits MERS-CoV infection	Albert et al., 2010; Kaplan et al., 2014; Kindrachuk et al., 2015
Selumetinib*	Blocks autophagy via specific inhibition of MEK1/MEK2	Inhibits MERS-CoV infection	Kindrachuk et al., 2015; Grasso et al., 2016
Venetoclax*	Activates autophagy by specifically inhibiting BCL2 and thus releasing Beclin-1 from inhibitory complex	Inhibits MERS-CoV replication	Malik et al., 2011; Gassen et al., 2019
3-Methyl Adenine (3-MA)	Inhibits autophagy by blocking autophagosome formation via the inhibition of class III PI3K	Decreases MHV and PEDV replication but not infection	Seglen and Gordon, 1982; Prentice et al., 2004a; Guo et al., 2017
Bafilomycin A1	Disrupts autophagic flux by inhibiting V-ATPase dependent autolysosomal acidification and autophagosome-lysosome fusion	Blocks PEDV entry into host cells	Yamamoto et al., 1998; Liu et al., 2016
Wortmannin	Suppresses autophagy via the inhibition of class III PI3K.	Inhibits MERS-CoV infection	Blommaart et al., 1997; Kindrachuk et al., 2015
UO126	Inhibits autophagy by blocking MAPK/ERK pathway	Inhibits MERS-CoV infection	Zhu et al., 2007; Kindrachuk et al., 2015
Valinomycin	Activates mitophagy by dissipating mitochondrial membrane potential and triggering complete removal of mitochondria; activates autophagy by stabilizing Beclin-1 via blocking Skp2 E3 ligase activity	Inhibits replication of SARS-CoV, MERS-CoV, HCoV-OC43, HCoV-NL63 and HCoV-229E	Wu et al., 2004; Smith and Blaylock, 2007; Rakovic et al., 2010; Gassen et al., 2019; Shen et al., 2019; Su et al., 2019; Sandler et al., 2020

*FDA approved drugs; PEDV, porcine epidemic diarrhea virus; HCoV-OC43, Human coronavirus OC43; HCoV-NL63, Human coronavirus NL63; HCoV-229E, Human coronavirus 229E.

## Chloroquine and Hydroxychloroquine

Although currently it remains highly debatable, two FDA (Food and Drug Administration) approved anti-malarial drugs CQ and its derivative hydroxychloroquine (HCQ) created a buzz for the treatment of COVID-19 (Bonam et al., 2020; Gao et al., 2020; Liu et al., 2020). CQ and HCQ belong to 4-aminoquinolines class of drugs, are weak bases, having half life of approximately 50 days (Schrezenmeier and Dorner, 2020). Beside anti-malarial effects, both CQ and HCQ also exhibit immunosuppressive functions, anti-inflammatory properties, as well as autophagy inhibitory roles (Gies et al., 2020; Schrezenmeier and Dorner, 2020). Accordingly, both drugs often used as anti-rheumatologic agents for the treatment of Systemic lupus erythematosus and rheumatoid arthritis and are classified as Disease-Modifying Antirheumatic Drugs (DMARDs) (Schrezenmeier and Dorner, 2020). Additionally, *in vitro* both drugs also demonstrate anti-viral properties by inhibiting autophagy pathway (Savarino et al., 2003; Keyaerts et al., 2004, 2009; Bhattacharjee et al., 2018). Clinically as compared to CQ, HCQ is a better drug in terms of safety index (Mcchesney, 1983; Liu et al., 2020). Although a number of mechanisms have been suggested for these drugs, the precise modes of action are yet to be defined.

Recently CQ and HCQ were shown to inhibit SARS-CoV-2 infection *in vitro* in Vero E6 cells (Liu et al., 2020; Wang et al., 2020). Although CQ is a lysosomotropic agent that inhibits autophagy-mediated degradation via blocking fusion between autophagosomes and lysosomes (Mauthe et al., 2018; Gies et al., 2020; Schrezenmeier and Dorner, 2020), its anti-autophagy activity may not be essentially underlying the antiviral activity. As discussed above, since the endosomal acidification plays a central role for CoV entry mechanism including SARS-CoV-2 (Yang and Shen, 2020), indeed CQ can limit the acidification and thereby blocking viral replication. Moreover, CQ restricts terminal glycosylation of the metallopeptidase ACE2 (Savarino et al., 2006), the functional receptor for viral entry into the host cells, which specifically interacts with SARS-CoV-2 spike protein whereas the nonglycosylated receptor appears to interact less efficiently (Zhao et al., 2020). However, since these functions are associated with the upstream of autophagy flux, and thus it is unlikely that CQ mediated inhibition of autophagy function may directly contribute to its antiviral activities. Furthermore, CQ exhibits several autophagy independent roles. For example, CQ is a potent pulmonary vasodilator that attenuates hypoxia−induced pulmonary hypertension (Wu et al., 2017). CQ also induces severe disorganization of the Golgi and endo-lysosomal systems, which might contribute to the fusion impairment (Mauthe et al., 2018). Altogether, these activities contribute to its much debatable therapeutic mechanism. Intriguingly, the overall effects of CQ on autophagy may fluctuate depending on a number of parameters, such as multiple cell types and drug amount. For example, the endosomal degradation process is catalyzed by two distinct proteases – (i) typically by TMPRSS2, a pH independent plasma membrane resident serine protease and (ii) sometimes by an endosomal-pH-dependent cysteine protease cathepsin L (CTSL). While TMPRSS2 activity is essential for viral entry and subsequent pathogenesis (Hoffmann et al., 2020a), CQ/HCQ mediated elevation of pH did not affect SARS-CoV-2 entry in lung cell line (Calu-3) expressing CTSL in very low concentration (Hoffmann et al., 2020b). However, by increasing intracellular pH, CQ and its structural analog HCQ can disrupt CTSL mediated viral entry in the African green monkey kidney cells (Vero E6), where the enzyme is abundantly expressed (Hoffmann et al., 2020b). In sum, these results imply that CQ and HCQ may not exert any antiviral activity in lung tissue of COVID-19 patients, as similar to the results of recent clinical trials (Boulware et al., 2020; Kupferschmidt, 2020). A number of meta-analyses collectively demonstrated that neither HCQ alone nor in combination with azithromycin, exhibited favorable treatment against COVID-19 patients (Fiolet et al., 2020). As a result, WHO (NCT04330690), NIH (National Institutes of Health, USA; NCT04358068) as well as pharmaceutical companies like Novartis (CJWT629A12301, NCT04358081) have discontinued their clinical trials of HCQ on COVID-19.

However, a number of research groups strongly believed potential therapeutic roles via modulation of autophagy pathway against SARS-CoV-2 infection. Below, we will discuss and highlight on such compounds either directly or indirectly affect autophagy pathway and thereby holding promise to use as an alternative approach to combat against SARS-CoV-2 infection.

## Rapamycin/Sirolimus

The mechanistic target of rapamycin (mTOR), a serine-threonine kinase, acts as a central regulator of cell metabolism, proliferation, and survival (Wullschleger et al., 2006; Laplante and Sabatini, 2009). In addition, mTOR also plays a key role in immune-regulation (Wullschleger et al., 2006; Dowling et al., 2010; Chen et al., 2019). It functions as one of the major intracellular nutrient sensors to control cell proliferation by sensing the extracellular energy state conferred by amino acids, glucose, growth factors, and hormones (Dunlop and Tee, 2009; Laplante and Sabatini, 2009; Dowling et al., 2010). Accordingly, under favorable growth conditions mTOR allows cell metabolism (viz. protein synthesis, lipids metabolism), whereas in response to nutrient deprived conditions mTOR inhibition activates catabolic processes (viz. autophagy) (Laplante and Sabatini, 2009). As a result, aberrant signaling of mTOR leads to various pathological conditions, including cancer, cardiovascular disease, inflammation, autoimmunity, and metabolic disorders (Dowling et al., 2010; Chong et al., 2011; Perl, 2015; Saxton and Sabatini, 2017; Hua et al., 2019). mTOR exists in two distinct multi-protein complexes - mTOR Complex 1 (mTORC1) and mTOR Complex 2 (mTORC2) (Laplante and Sabatini, 2009; Dowling et al., 2010). Sirolimus, also known as rapamycin, a FDA-approved immunosuppresive drug, typically inhibits the mTORC1 activity (Albert et al., 2010). The rapamycin−sensitive mTORC1 primarily functions as a nutrient or energy sensor and thereby regulating protein synthesis, adipogenesis, ribosome biogenesis and mitochondrial metabolism. On the other hand, mTORC2 regulates cell proliferation and survival, actin cytoskeleton and ion transport (Dunlop and Tee, 2009; Laplante and Sabatini, 2009; Dowling et al., 2010).

Accumulating evidence suggests mTORC1 plays a key role in regulating replication of a number of viruses (Bowman et al., 2018; Maiese, 2020). For example, mTORC1 positively regulates replication of a New World hantavirus - Andes virus (ANDV), as treatment with a FDA-approved rapamycin analog temsirolimus (CCI-779) significantly inhibits viral protein expression and virion release (McNulty et al., 2013). In contrast, mTORC1 blocks hepatitis C virus (HCV) replication through Unc-51 like autophagy activating kinase ULK1 activation, but facilitates virion packaging and subsequent release (Johri et al., 2020). In addition, adjuvant treatment with sirolimus in combination with corticosteroids revealed better clinical outcomes in patients with influenza A virus subtype H1N1 mediated severe pneumonia and acute respiratory failure (Wang et al., 2014). Moreover, sirolimus has also been shown to have antiviral effects against CoVs. Sirolimus treatment decreased MERS-CoV infection by approximately 60%, providing a strong evidence for a critical role of mTOR in CoV pathogenesis (Dyall et al., 2017). Recently using network proximity analyses of drug targets as well as CoV–host interactions in the human interactome, sirolimus in combination with dactinomycin (actinomycin D, is a FDA approved RNA synthesis inhibitor) has been suggested as potential drug candidates for the treatment of COVID-19 patients (Zhou et al., 2020b). As discussed above, a recent preprint demonstrated that SARS-CoV-2 infection reprograms the metabolism of cells by limiting AMPK/mTORC1 activation and autophagy flux, which in turn facilitates viral replication (Gassen et al., 2020). Consequently, mTORC1 inhibition by rapamycin promotes virus growth (Gassen et al., 2020). Although rapamycin has been proposed as a potential treatment option for COVID-19, these results indicate that comprehensive molecular and functional analyses need to be carried out before considering its clinical use. However, considering the potent anti-viral properties and prior FDA-approval, it is highly recommended to conduct randomized, double−blinded, placebo−controlled, multicenter clinical trials to validate the safety and efficacy of mTOR inhibitors to combat COVID−19 severity. In fact, currently multiple small-scale clinical trials (NCT04341675; NCT04371640; NCT04461340) are underway to determine sirolimus as a potential treatment option in COVID-19 patients.

## Everolimus

In line with its antiviral effectiveness and other potential therapeutic exercises along with its poor solubility and pharmacokinetics, a number of structural analogs (rapalogs) of sirolimus have been synthesized (Albert et al., 2010; Schreiber et al., 2019). Among these, everolimus represents as one of the FDA-approved promising second-generation sirolimus derivatives at least in terms of effectively managing the adverse events caused by rapamycin inhibitors in solid-organ transplant recipients as immunosuppressive agents along with in treatment of various cancers especially advanced renal cell carcinoma (Kaplan et al., 2014; Klawitter et al., 2015). In addition, everolimus has a greater bioavailability than sirolimus and it decreases vascular inflammation, more so than sirolimus (Klawitter et al., 2015). Furthermore, everolimus was shown to inhibit replication of a number of oncogenic viruses including human cytomegalovirus (HCMV), BK virus (BKV), hepatitis C virus (HCV) and Epstein-Barr virus (EBV) in post-transplant and cancer patients (Garofalo et al., 2019; Nanmoku et al., 2019; Tan et al., 2019). As discussed above, accumulating evidence suggests mTOR inhibition and subsequent autophagy activation as an attractive therapeutic option in managing the ongoing COVID-19 pandemic. Indeed, as similar to the classical mTOR inhibitor sirolimus, everolimus can also radically reduce MERS-CoV infection (Kindrachuk et al., 2015). However, mTOR inhibitors also functions as powerful immunosuppressant by decreasing proliferation of conventional T-lymphocytes that recognize peptide antigens presented by MHC molecules, while inducing proliferation of T-regulatory cells (Tregs), which suppresses other immune cells (Wullschleger et al., 2006; Dowling et al., 2010; Chen et al., 2019). Therefore, everolimus mediated inhibition of mTOR activity may exert differential outcomes in COVID-19 patients by mitigating the cytokine storm and hyper-reactivity in the critical phase of the disease (Terrazzano et al., 2020; Ye et al., 2020). Overall, the beneficial effects of everolimus on mTOR-mediated autophagy activation must be cautiously evaluated alongside with its effects on host immune system, when treating COVID-19 patients. It has been shown that everolimus at high doses acts as an effective immunosuppressive agent, while at lower doses it accelerates the host immune response (Mannick et al., 2014). Thus, everolimus needs to be administered in case of COVID-19 patients at higher doses as parallel to the dosage used for organ transplant recipients and cancer patients.

## Trehalose

Trehalose, a naturally occurring non-reducing disaccharide found in plants, insects, microorganisms, and invertebrates, demonstrates cytoprotective properties against various neurodegenerative diseases and several metabolic disorders through activating autophagy (Yoon et al., 2017). Trehalose induces autophagy independently of mTOR inhibition and first reported to facilitate aggrephagy in neuronal cells (Sarkar et al., 2007). However, the exact mechanism of trehalose mediated induction of autophagy remains debatable, a number of potential mechanisms have been recently suggested. For example, trehalose reduces AKT mediated phosphorylation of one of the primary lysosomal biogenesis regulator, transcription factor EB (TFEB), and thus enabling its translocation to the nucleus independently of the TFEB inhibitor, mTOR (Sardiello et al., 2009; Palmieri et al., 2017). A number of clinical trials are currently underway to validate trehalose as an anti-inflammatory agent in patients suffering from vascular inflammation and atherosclerosis (NCT 03700424) as well as to modulate endothelial function, oxidative stress, platelet function, and autophagy against peripheral arterial disease (NCT 04061070). Additionally, in the context of viral diseases, trehalose also exhibits potent anti-viral functions through modulating autophagy. For instance, trehalose augments virophagy against HIV infection and thereby reducing viral load in peripheral blood mononuclear cells (PBMCs) isolated from AIDS patients (Sharma et al., 2020). Trehalose was also shown to block replication of both human cytomegalovirus (HCMV) and varicella-zoster virus (VZV) *in vitro* through autophagic induction (Buckingham et al., 2014; Belzile et al., 2015). Although there is no direct evidence of trehalose mediated inhibition of CoV replication, given the critical role of endocytosis in viral entry mechanism (Yang and Shen, 2020), it would be fascinating to investigate its potential inhibitory effect on SARS-CoV-2 infection. Owing to its antiviral properties against many viruses as well as falling into the FDA approved drug categories, trehalose can be used as a prophylactic measure as an alternative approach to combat the current COVID-19 outbreak.

## Valinomycin

Valinomycin, a cyclodepsipeptide antibiotic that selectively induces the transport of potassium ion (K ^+^) across the membrane, and to date, the function of valinomycin has been elucidated in Parkin−/PINK1 regulated mitophagy (Narendra et al., 2010; Rakovic et al., 2019; Su et al., 2019). In the presence of valinomycin, mitochondria absorb K ^+^ at the expense of the proton (H ^+^)-gradient, ensuing in dissipation of membrane potential (Smith and Blaylock, 2007; Joshi and Bakowska, 2011). Alike CCCP (Carbonyl cyanide m-chlorophenyl hydrazone), an uncoupler of mitochondrial oxidative phosphorylation, valinomycin can also accelerate PINK1 accumulation and Parkin translocation (Narendra et al., 2010; Rakovic et al., 2010). It has broad spectrum potential therapeutic functions exhibiting anticancer, antibacterial, antifungal as well as antiviral properties (Pettit et al., 1999; Wu et al., 2004; Cheng, 2006; Iacobazzi et al., 2013; Zhang et al., 2020a). The first report supporting valinomycin as one of the compelling anti-CoV compounds was based on screening of more than ten thousand small molecule drug candidates that effectively block SARS-CoV replication (Wu et al., 2004). However, the mechanism by which valinomycin inhibits SARS-CoV replication is not yet known. Subsequently, accumulating evidence indicates its potential antiviral activity against three other human CoVs including MERS-CoV, HCoV-OC43 and HCoV-NL63 along with a murine CoV MHV-A59 (Gassen et al., 2019; Shen et al., 2019; Sandler et al., 2020). Valinomycin, which is also known to function as Skp2 E3 ligase inhibitor, was recently shown to block MERS-CoV replication by activating autophagy flux through stabilizing Beclin-1 (Gassen et al., 2019). In agreement to this, a high-throughput screening (HTS) of a 2000-compound library comprising of both FDA-approved drugs and pharmacologically active small molecules demonstrated inhibitory effects of valinomycin on MERS-CoV along with MHV-A59 and two other human α-CoVs including HCoV-OC43 and HCoV-NL63 (Shen et al., 2019). In an attempt to identify potential antiviral compounds against the La Crosse virus (LACV) using the NIH’s Developmental Therapeutics Program (DTP) containing more than 500 compounds, valinomycin made as one of the top hit compounds, exhibiting antiviral activity against several other viruses including two CoVs, MERS-CoV and HCoV-229E (Sandler et al., 2020).

Of note, the genome of SARS-CoV-2 shares approximately 80% and 50% sequence identity to SARS-CoV and MERS-CoV, respectively (Hu et al., 2020). Moreover, as similar to SARS-CoV and HCoV-NL63, SARS-CoV-2 also utilizes the ACE2 cell surface receptor for host cell entry mechanism (Hoffmann et al., 2020a; Zhao et al., 2020). Given its potent antiviral property specifically against several CoVs, valinomycin holds merit as potential drug in the COVID-19 treatment regimen. Future work demands modifications of the structure to reduce cytotoxicity while maintaining the antiviral activities of the synthesized derivatives of valinomycin.

## Conclusion and Future Perspective

Till date, there is no specific treatment for patients suffering from COVID-19. Moreover, due to the widespread infection, mutation rate of SARS-CoV-2 is significantly high. The pervasive threat of the disease has thus spawned challenges to develop safe and effective vaccines, has led to the development of over 300 vaccine projects including whole virus vaccines, live attenuated vaccines, recombinant protein subunit vaccines, and nucleic acid vaccines. Among these, more than 40 different vaccines are currently underway clinical evaluation and 3 out of 10 have already ended Phase III clinical trials with encouraging results (Hodgson et al., 2020; Forni and Mantovani, 2021). Moreover, considering the current crisis situation, a number of these novel vaccines are being approved. For example, Covaxin based on inactivated SARS-CoV-2 virion, is currently in late stage of Phase III clinical trial by Bharat Biotech, India, has already been approved for mass vaccination in a highly populated country like India based on the results from Phase II clinical trial indicating that the vaccine is safe and can induce a high titre of antibodies (Forni and Mantovani, 2021). Due to short development time there are several unanswered facts which need to be clarified whether and to what extent these vaccine candidates enhance immunity against SARS-CoV-2 infection. In the long run, therefore we need more than one vaccine ensuring an unbiased global access particularly in the underdeveloped countries, protection of diverse population of different ethnic background as well as immunity against multiple viral strains. In addition to boost host innate immunity, among various strategies employed, passive immunotherapy using camelid-derived single domain antibody fragments, also known as VHH or nanobodies, are of great interest for the treatment and prevention of COVID-19 (Hanke et al., 2020; Lu et al., 2021). Although retaining specificity and affinity, due to their substantial smaller size, around one tenth of the conventional antibodies, nanobodies possess several advantages, like easy manipulation, cloning, express in bacteria in large quantities along with a number of favourable biochemical properties, including high thermostability and deep tissue penetration (Hanke et al., 2020; Koenig et al., 2021). Importantly, nanobodies have previously been demonstrated as potent anti-viral agents particularly against respiratory infections (Cardoso et al., 2014; Detalle et al., 2016; Cunningham et al., 2021). Similarly, several efforts are currently underway generating both monovalent and multivalent nanobodies particularly targeting the receptor binding domain of the viral spike protein and thereby competing with ACE2 binding (Barnes et al., 2020; Lv et al., 2020; Rogers et al., 2020; Walls et al., 2020; Koenig et al., 2021). Of note, recently Koenig et al. developed structure guided multivalent nanobodies that specifically inhibit SARS-CoV-2 infection as well as block mutational escape (Koenig et al., 2021). Overall, humanized multivalent nanobodies in form of nasal spray show great promise in order to prevent viral spread particularly among vulnerable population.

Although there are several novel small molecule compounds are currently under clinical trials, more randomized controlled trials (RCT) need to be urgently conducted before any of these drugs become available to the public without any severe toxicity issues. Hence, a faster, safer and cost-effective approach is necessary in order to restrain the viral spread. To this end, repurposing of FDA-approved drugs can bring treatment to COVID-19 patients with much faster and less cost as compared to developing new drugs. So far, 3807 clinical trials regarding COVID-19 are in progress^2^, where most of the studied compounds are repurposed drugs. For example, simvastatin, or statins in general, are currently under clinical trial (NCT04407273) as potential treatment strategy of COVID-19 patients based on their role in regulating inflammatory response induced by SARS-CoV-2 infection and in hospital use of statins with reduced mortality in COVID-19 patients (Garcia, 2020; Rossi et al., 2020; Zhang et al., 2020b).

Notably, a number of these FDA-approved compounds including CQ and its structural analog HCQ, baricitinib, dexamethasone, as well as remdesivir have initially been given emergency approval for the treatment of COVID-19 patients (Beigel et al., 2020; Boulware et al., 2020; Cantini et al., 2020; Gao et al., 2020; Horby et al., 2020a). Artificial intelligence also plays an important factor in finding the appropriate drug for disease management (Zhou et al., 2020b,c). For example, as discussed above, analysis of ∼81000 human genomes suggested that both CQ and HCQ failed to work in TMPRSS2-positive COVID-19 patients (Hou et al., 2020). In agreement to this, CQ is unable to inhibit SARS-CoV-2 infection of the TMPRSS2-positive lung cell line Calu-3, whereas HCQ exhibits antiviral activity in Vero E6 cells without TMPRSS2 expression (Hoffmann et al., 2020b). Similarly, another study showed HCQ exhibited antiviral activity in Vero E6 cells but not in a model of reconstituted human airway epithelium (Maisonnasse et al., 2020). Additionally, the same group also argued the use of HCQ, either alone or in combination with azithromycin for the treatment of COVID-19 patients based on their results using SARS-CoV-2 infected non-human primates, macaques (Maisonnasse et al., 2020). In contrast, in a pilot observational study of 80 COVID-19 patients followed by a non-randomized clinical trial of 20 patients of upper respiratory tract infection symptoms, HCQ treatment significantly declined the viral load and its efficacy was further enhanced in the presence of azithromycin (Gautret et al., 2020a,b). Although three randomized controlled trials of CQ/HCQ against COVID-19 patients reported earlier significant favourable outcome in terms of extent of cough and fever, withdrawal of clinical symptoms, declining in overall patients’ death and viral shedding (Chen et al., 2020; Million et al., 2020; Sarma et al., 2020), later controversial results from different clinical trials of HCQ alone or in combination with azithromycin (Fiolet et al., 2020; Horby et al., 2020b; Self et al., 2020) led WHO to discontinue their “Solidarity” clinical trial (NCT04330690) for COVID-19 treatments (Burki, 2020). It was one of the largest international randomized trials for COVID-19 treatments, enrolling almost 10000 patients in 500 hospital sites across 27 countries with an aim to evaluate the effect of drugs on three critical outcomes of patients that includes mortality, ventilation requirement as well as and duration of hospital stay (Burki, 2020). HCQ can cause severe hypoglycaemia when co-administered with oral hypoglycaemic drugs (Oscanoa et al., 2020; Rosenberg et al., 2020). A number of studies also reported HCQ treatment results in mild to severe hepatic failure in COVID-19 patients (Taccone et al., 2020). In addition, cytotoxicity associated with racial difference has also been reported wherein the incidence of pericentral retinopathy appears common in Asian population (50%) as compared to Caucasian patients (2%) (Melles and Marmor, 2020).

Nevertheless, successful execution of an autophagy modulator as a safe and efficacious therapy against COVID-19 patients warrants further investigation in order to increase its specificity at the site of action (infected cells), which in turn alleviates its off-target effects. Altogether, this groundwork highlights the significance of pharmacogenomics studies in improving clinical benefits as well as the success rate of drug repurposing in COVID-19 patients. Recent genome-wide association studies identified a gene cluster on chromosome 3 as a major genetic risk factor for respiratory failure and other associated severity in COVID-19 patients (Ellinghaus et al., 2020; Zeberg and Paabo, 2020). Overall, locating human genetic determinants of viral pathogenesis may thus provide a unique opportunity for drug repurposing in personalized treatment for individual COVID-19 patient. In addition, based on the current knowledge available (Gassen et al., 2020; Singh et al., 2020), we strongly believe autophagy inducers (such as Sirolimus, Everolimus, Trehalose and Valinomycin) hold great promise as an alternative therapeutic strategy. Overall, the magnitude of both drug repositioning as well as preclinical evaluation of drug combinations should be taken into consideration for potential therapeutic use against SARS-CoV-2 infection.

## Author Contributions

SM and AS wrote the manuscript and performed the literature search. AS designed the figures and conceived the idea, revised the manuscript, coordinated the activity, and concurred with the final version of the review. Both authors reviewed and approved the manuscript.

## Conflict of Interest

The authors declare that the research was conducted in the absence of any commercial or financial relationships that could be construed as a potential conflict of interest.

## Footnotes

1 www.worldometers.info/coronavirus/

2 www.clinicaltrials.gov/
